# The effect of aspirin on lumbar degeneration: an imaging-based study

**DOI:** 10.3389/fsurg.2024.1515585

**Published:** 2024-12-19

**Authors:** Haiyun Niu, Feiyu Zu, Zhenguo Shang, Ze Gao, Dazhuang Miao, Di Zhang

**Affiliations:** ^1^ Department of Spine Surgery, Hebei Medical University Third Hospital, Shijiazhuang City, Hebei, China; ^2^ Department of Joint Surgery, Hebei Medical University Third Hospital, Shijiazhuang City, Hebei, China

**Keywords:** aspirin, lumbar degeneration, spine, MRI, low back pain

## Abstract

**Purpose:**

This study aims to investigate how aspirin influences lumbar degeneration by analyzing the effect of aspirin on patients with low back pain (LBP) and concurrent atherosclerosis.

**Methods:**

Using 1:1 nearest neighbor matching based on propensity score matching (PSM), 73 patients who regularly took aspirin were assigned to the aspirin group, while another 73 patients who did not take aspirin formed the control group. Radiographs were used to measure lumbar lordosis (LL) and intervertebral height index (IHI). Subcutaneous fat tissue thickness (SFTT), paravertebral muscle fat infiltration area (%FIA), cartilage endplate (CEP) Modic changes, and modified Pfirrmann grading scores were performed based on lumbar MRI.

**Results:**

After PSM analysis, confounders between the aspirin and control groups were balanced. A total of 73 pairs of patients were analyzed in this study. The aspirin group showed lower SFTT(L1/2) and a reduced incidence of CEP Modic changes, compared to the control group (both *P* < 0.05). Additionally, the %FIA and Pfirrmann scores were lower in the aspirin group, particularly in the upper lumbar spine (both *P* < 0.05). No significant differences were observed in LL and IHI between the aspirin and control groups.

**Conclusion:**

In summary, conservative treatment with aspirin protects against upper lumbar spine degeneration, although its effect on the lower lumbar spine is less pronounced.

## Introduction

Lower back pain (LBP) is a widespread health issue globally, with a high incidence rate and most patients not requiring surgical treatment (1). Intervertebral disc degeneration (IVDD) is a significant cause of LBP (2). Approximately 80% of individuals will experience varying degrees of lower back pain at some point in their lives, and this condition is often associated with persistent pain that significantly impacts the patient's quality of life (3). The pain not only limits daily activities but may also lead to mental health problems such as depression and anxiety, creating a vicious cycle (4, 5). Therefore, exploring effective treatment options is particularly important, especially for chronic lower back pain patients with recurrent symptoms.

Currently, nonsteroidal anti-inflammatory drugs (NSAIDs) are widely used in clinical practice as the main symptomatic treatment for lower back pain. They are effective in alleviating pain, reducing inflammation, and improving the quality of life for patients (6, 7). However, some experimental researches indicate that NSAIDs, through their effects on inhibiting ferroptosis and inflammation, may help alleviate intervertebral disc degeneration to some extent (8, 9). Despite this, there is still a lack of systematic clinical studies in this area, and the long-term impact of NSAIDs in chronic pain management, especially whether they can fundamentally improve the pathological changes in lower back pain, is not well understood. Therefore, investigating whether long-term use of NSAIDs can slow the progression of lumbar degeneration is of great significance in developing more effective treatment strategies.

It is important to note that long-term use of NSAIDs may lead to serious side effects, such as gastrointestinal ulcers, cardiovascular diseases, and renal dysfunction, which limit their application in chronic lower back pain patients (10). Research on the time-dependent effects of NSAIDs is relatively scarce. Aspirin, as a type of NSAID, has been used long-term in patients with atherosclerosis due to its anti-platelet aggregation effect (11), addressing the problem of poor adherence to long-term medication caused by the side effects of NSAIDs. This study focuses on patients with lower back pain accompanied by atherosclerosis and divides them into groups based on their aspirin treatment regimen. By comparing the lumbar imaging changes between these two groups, the study aims to explore whether time-dependent aspirin use can provide fundamental benefits to patients. Through the analysis of imaging data, we hope to reveal the true value of NSAIDs in the treatment of chronic lower back pain, providing scientific evidence for clinicians to optimize pain management while maximizing the protection of patients' lumbar health and ensuring the sustainability of treatment outcomes.

## Materials and methods

### Study subjects, inclusion and exclusion criteria

**Figure 1:**
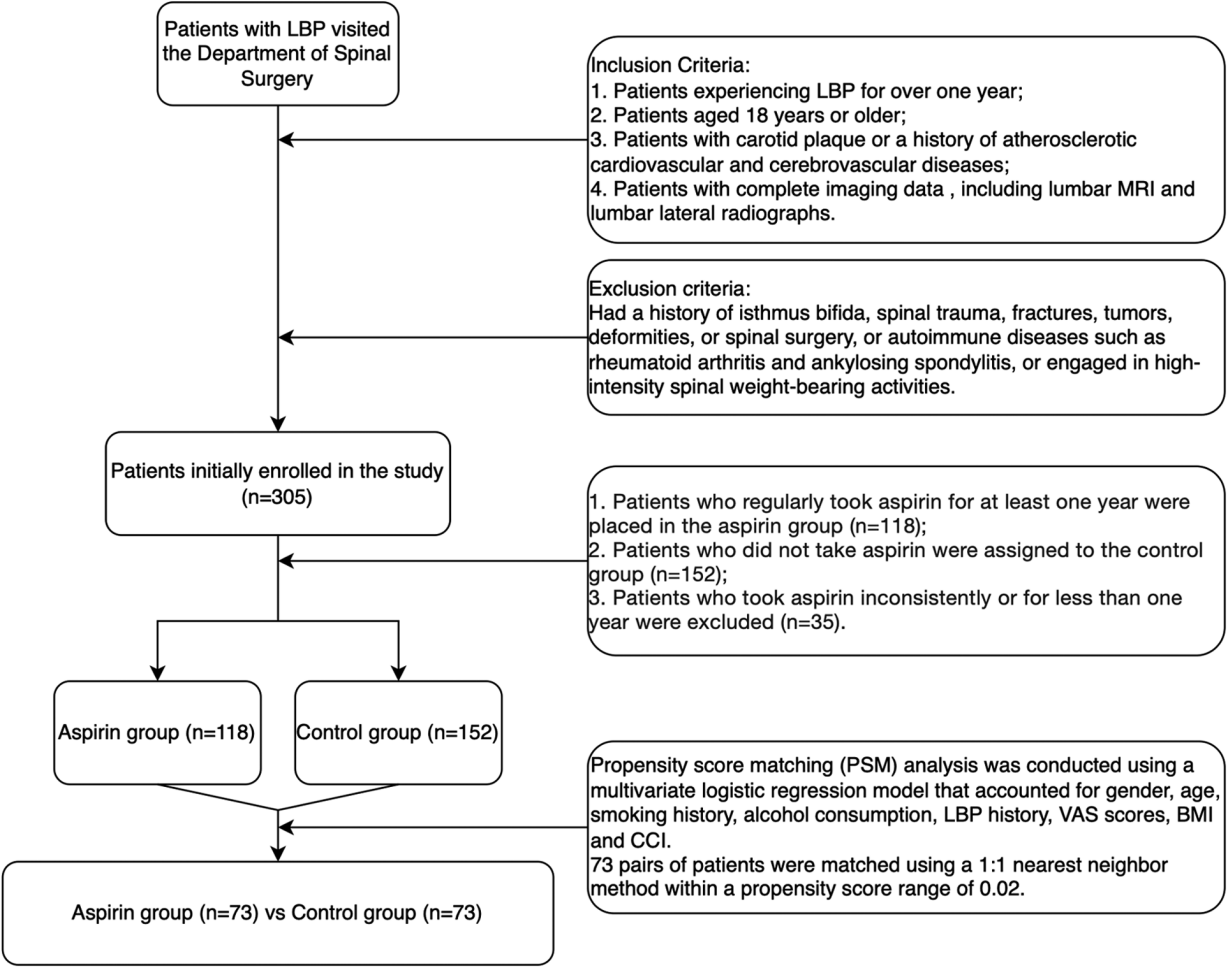
Flowchart of patient selection.

The study participants consisted of patients who visited the Department of Spine Surgery for LBP between January 1, 2023, and December 31, 2023 (Figure 1).

#### Inclusion criteria

1. Patients experiencing LBP for over one year.
2. Patients aged 18 years or older.
3. Patients with carotid plaques or a history of atherosclerotic cardiovascular or cerebrovascular diseases.
4. Patients with complete imaging data, including lumbar MRI and lateral radiographs.

#### Exclusion criteria

Patients were excluded if they had a history of isthmus bifida, spinal trauma, fractures, tumors, deformities, spinal surgeries, or autoimmune disorders such as rheumatoid arthritis and ankylosing spondylitis. Additionally, individuals engaged in high-intensity spinal weight-bearing activities were also excluded. Initially, 305 patients were enrolled in the study.

#### Grouping criteria

1. Patients who regularly took aspirin for at least one year were placed in the aspirin group (*n* = 118).
2. Patients who did not take aspirin were assigned to the control group (*n* = 152).
3. Patients who took aspirin inconsistently or for less than one year were excluded from both groups (*n* = 35).

The final analysis included 118 patients in the aspirin group and 152 patients in the control group. Propensity score matching (PSM) analysis was conducted using a multivariate logistic regression model that accounted for gender, age, smoking history, alcohol consumption, history of LBP, visual analogue scale (VAS) scores, body mass index (BMI), and the Charlson comorbidity index (CCI). A total of 73 pairs of patients were matched using a 1:1 nearest-neighbor method within a propensity score range of 0.02.

## Data collection

The data collected from the study participants encompassed various factors, including gender, age, history of LBP, smoking habits, alcohol consumption, VAS scores, BMI, CCI, SFTT (L1/2), fatty infiltration area ratio (%FIA) of paravertebral muscles, lumbar lordosis (LL), scores from the modified Pfirrmann grading system, intervertebral height index (IHI), incidence of Modic changes in the lumbar region, as well as results from lumbar MRI and lateral radiographs.

## Lumbar lordosis

**Figure 2:**
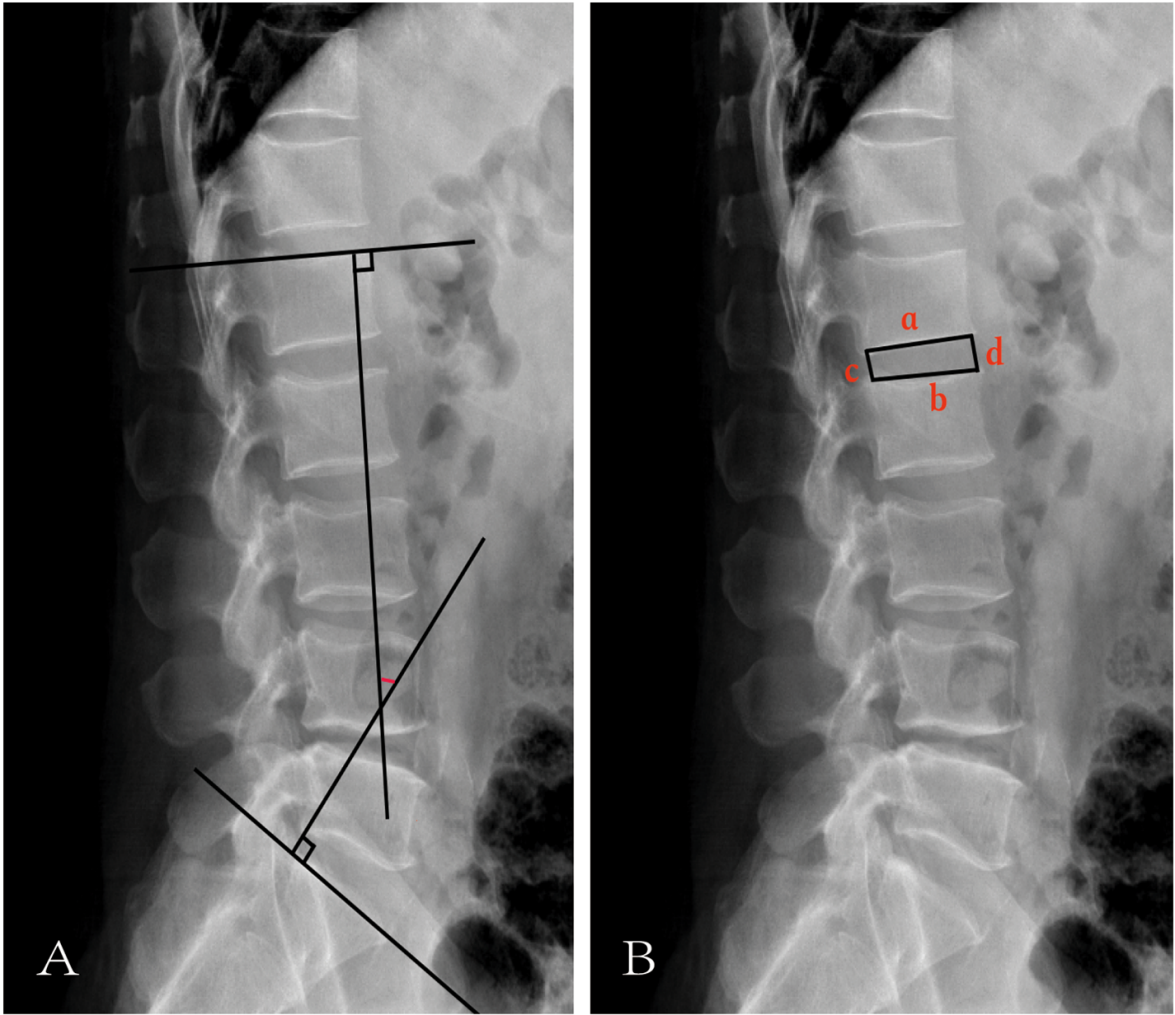
Illustration of LL and IHI. **(A)** The red mark represents LL. **(B)** **a** represents the length of the lower edge of the upper vertebrae. **b** represents the length of the upper edge of the lower vertebral body. **c** represents the height of the posterior edge of the intervertebral space. d represents the height of the anterior edge of the intervertebral space.

Lumbar lordosis was assessed using a standard lumbar lateral radiograph by calculating the angle formed between the tangents of the upper endplates of the L1 and S1 vertebrae (12) (Figure 2A).

## Intervertebral height index

As illustrated in Figure 2B, IHI was calculated using the following formula (13):

$$\mathrm{IHI}=\frac{d+c}{a+b}\;\times \;100\text{\%}$$

*a*: length of the lower edge of the upper vertebrae,
*b*: length of the upper edge of the lower vertebral body,
*c*: height of the posterior edge of the intervertebral space,
*d*: height of the anterior edge of the intervertebral space.

## Modified Pfirrmann grading scores

The modified Pfirrmann grading system (14) was used to assess the grade of IVDD based on MRI images. Specifically, T2-weighted images from the central sagittal MRI of the lumbar spine were utilized. A senior spinal surgeon and a senior radiologist independently evaluated the IVDD in each patient using the modified Pfirrmann grading system. Both evaluators were blinded to the study design. In cases where their assessments differed, discrepancies were resolved through discussion and consensus.

## Subcutaneous fat tissue thickness(L1/2)

**Figure 3:**
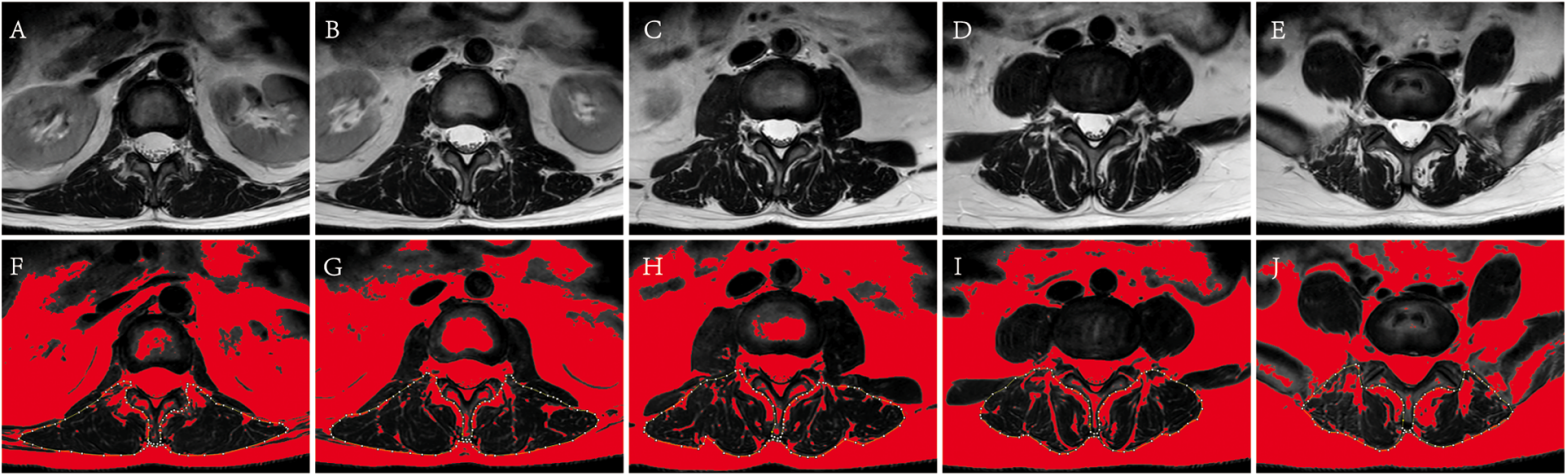
The measurement of paravertebral muscle %FIA of L1-S1. **(A–E)** Correspond to original cross-section images of L1/2, L2/3, L3/4, L4/5, L5/S1. **(F–J)** Correspond to paravertebral muscle %FIA images of L1-S1 by ImageJ management. The fat tissue was displayed in red, and the yellow dotted line showed the bilateral paravertebral muscles using the threshold technique.

In the sagittal image of the lumbar MRI, the L1/2 segment was located. In the corresponding cross-sectional image of the lumbar MRI, the vertical distance from the tip of the L1 spinous process to the skin was measured (Figure 3), which was the SFTT(L1/2) (15). All measurements were calculated using the RadiAnt DICOM Viewer (Version 2020.1, Medixant).

## Paravertebral muscles fat infiltration area ratio

**Figure 4:**
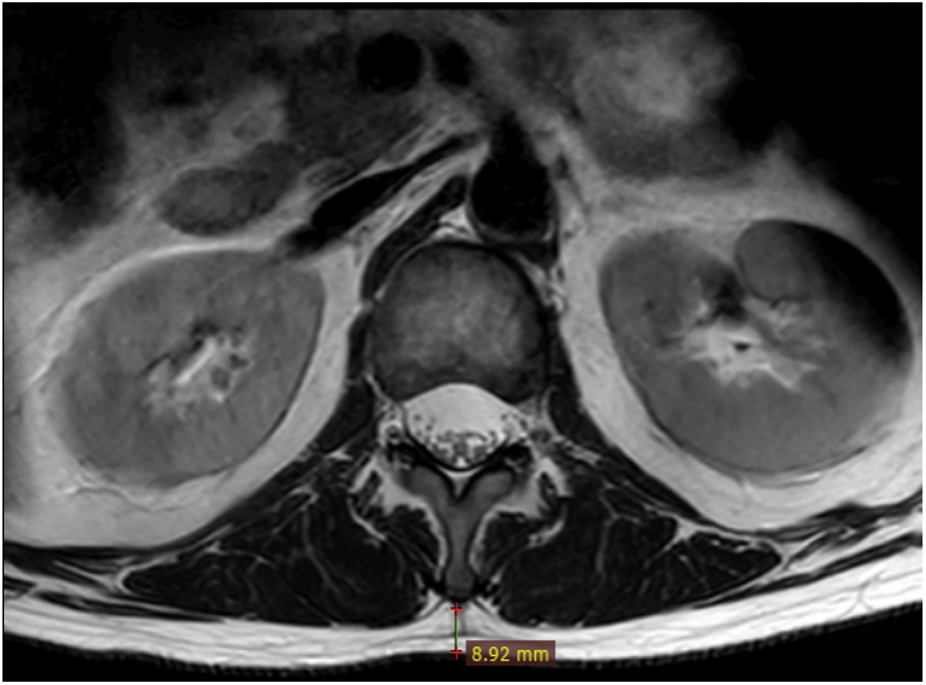
Measurement technique of SFTT(L1/2). At the L1/2 level on T2-weighted axial lumbar MRI, the vertical distance from the L1 spinous process to the skin was SFTT(L1/2).

The median position of the L1-S1 intervertebral disc was identified on the sagittal images from lumbar MRI, and the paravertebral muscles %FIA was measured using cross-sectional images of the corresponding segments (16). Firstly, the selected image was converted into a grayscale 8-bit image by ImageJ software. Secondly, the bilateral paravertebral muscles region was delineated along the paravertebral muscles boundary with bare hands, and selected. Thirdly, selected image/adjust/threshold/dark background and default/auto in order on the Image J software, the adipose tissue was displayed in red, and the Image J software could directly calculate the percentage of adipose tissue in the selected area, which was the paravertebral muscles %FIA (Figure 4). All the data was calculated by image J (Version 1.53k, Wayne Rasband, NIH, USA).

## Incidence of cartilage endplate Modic changes

**Figure 5:**
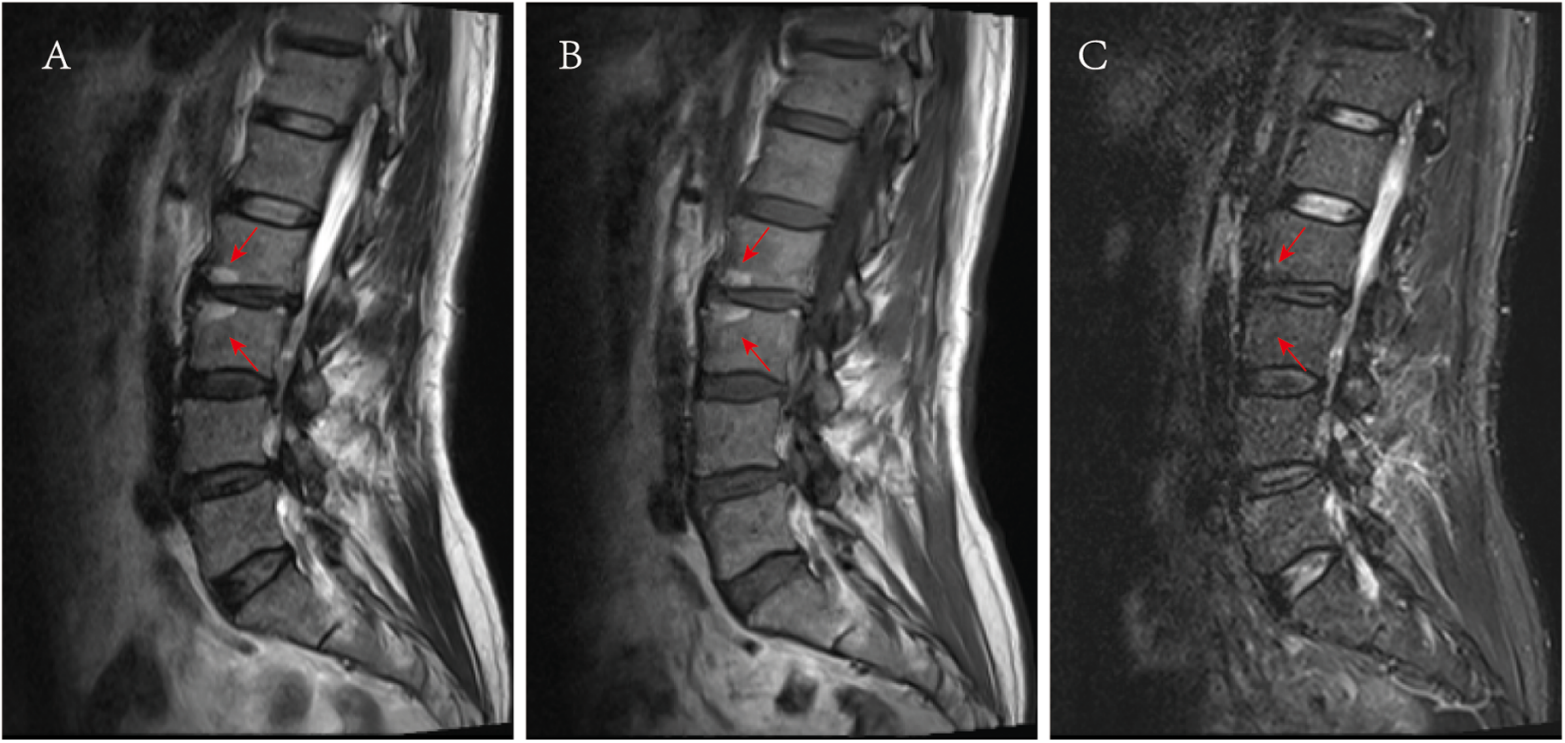
Images of Modic II. **(A)** Presented a high signal on the T2WI sagittal MRI, as shown by the red arrow. **(B)** Presented a high signal on the T1WI sagittal MRI, as shown by the red arrow, **(C)** A low signal was presented on the sagittal at fat suppression sequence MRI, as shown by the red arrow.

Count the number of CEP Modic changes based on the following criteria:

Modic I: T1-weighted images (T1WI) show low signal, T2-weighted images (T2WI) exhibit high signal, and fat suppression sequences display high signal.
Modic II (Figure 5): T1WI shows a high signal, T2WI shows a slightly high signal and fat suppression sequences demonstrate a low signal.
Modic III: T1WI, T2WI, and fat suppression sequences all display low signals.

Each patient has a total of 10 cartilage endplates, ranging from the lower cartilage endplate of L1 to the upper cartilage endplate of S1. The incidence of CEP Modic changes is calculated as the total number of CEP Modic changes divided by the total number of CEPs.

## Consistency of measurements and ratings

To ensure measurements and rating consistency, Cohen's kappa analysis was conducted for pathological changes of the CEPs (0.902), weighted kappa analysis was used for disc degeneration grading (0.823), and intraclass correlation efficient (ICC) analysis was applied for LL (0.912), IHI (0.906), SFTT (0.935) and %FIA (0.928), respectively.

## Statistical analysis

All data were analyzed by SPSS 27.0 (IBM, Armonk, New York, United States) or R Project (Version 4.4.1, CRAN). The R Project was utilized for PSM analysis. The Kolmogorov-Smirnov test was employed to assess the normality of data distribution. Data with a normal distribution were presented as mean ± standard deviation, while non-normally distributed data were reported as median and interquartile range (IQR). For comparisons of continuous variables between groups, an independent sample *t*-test was applied to normally distributed data, and the Mann–Whitney *U*-test was used for non-normally distributed data. Categorical variables were analyzed using the chi-square test. A *p*-value of less than 0.05 was considered statistically significant.

## Results

### Match confounders by propensity score matching

**Figure 6:**
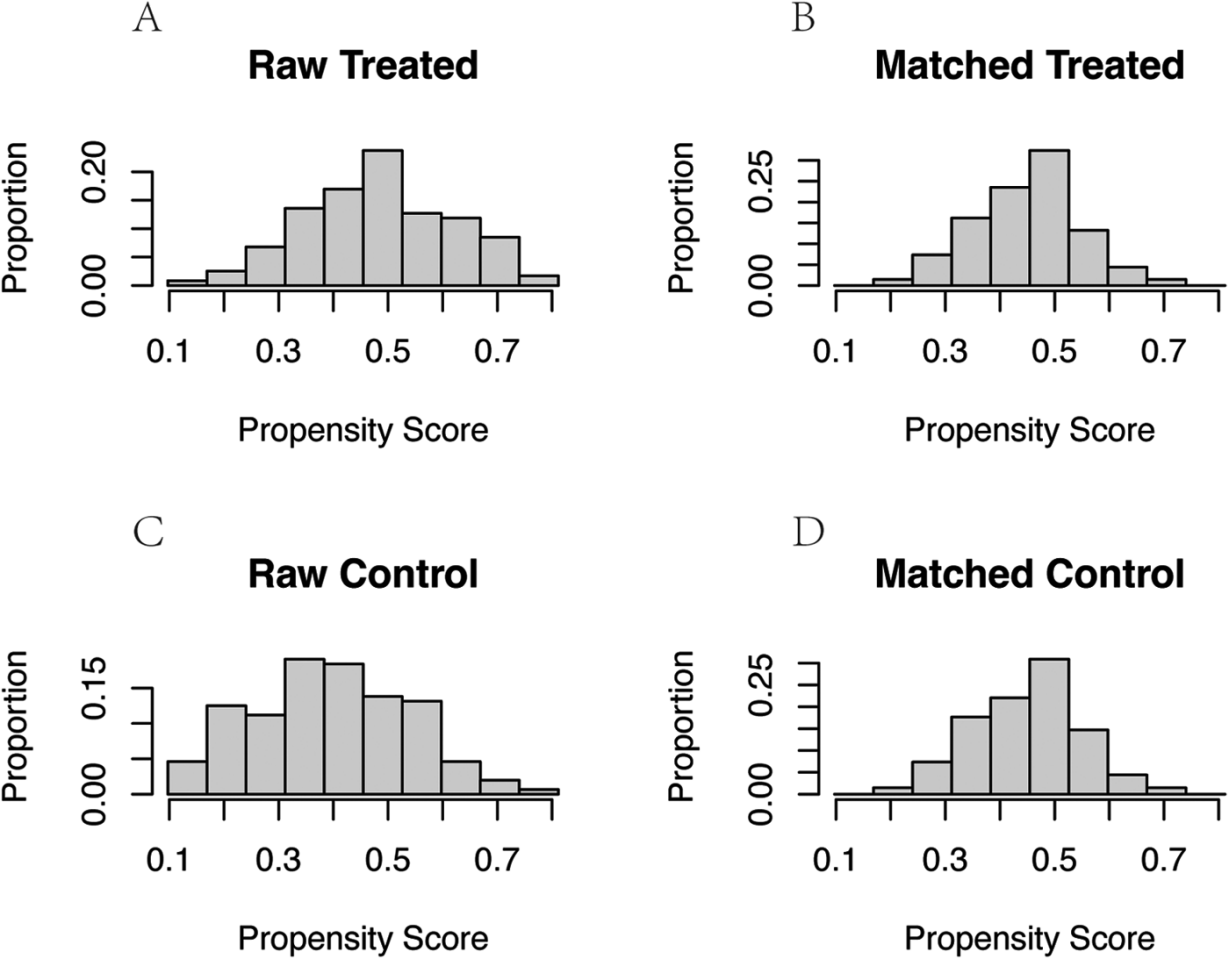
Propensity score and proportion of confounders before and after PSM. **(A)** Propensity score and proportion of confounders before PSM in aspirin group. **(B)** Propensity score and proportion of confounders after PSM in aspirin group. **(C)** Propensity score and proportion of confounders before PSM in control group. **(D)** Propensity score and proportion of confounders after PSM in control group.

Through PSM analysis (Figure 6), confounding factors such as gender, age, history of LBP, smoking history, alcohol consumption, VAS score, BMI, and CCI were matched (Table 1). A total of 146 patients were included in the study using 1:1 nearest neighbor matching within a propensity score range of 0.02 (Table 2). General clinical characteristics (gender, smoking history, alcohol consumption) were compared using the chi-square test, and no significant differences were observed between the aspirin and control groups (*p* > 0.05). The average age in the aspirin group was 63.09 ± 7.48 years, while the control group had an average age of 62.57 ± 7.53 years (*p* = 0.69). The aspirin group had an average LBP history of 42.26 ± 50.10 months, compared to 45.15 ± 64.49 months in the control group (*p* = 0.771). The mean BMI in the aspirin group was 25.70 ± 3.50 kg/m^2^, similar to the control group's 25.72 ± 3.27 kg/m^2^ (*p* = 0.968). The average VAS score was 5.85 ± 1.76 in the aspirin group and 5.90 ± 1.64 in the control group (*p* = 0.88). The mean CCI was identical in both groups, at 2.13 ± 1.09 for the aspirin group and 2.13 ± 0.99 for the control group (*p* = 1.00).

**Table 1:** Comparison of confounders before PSM.

	Control group (*n* = 152)	Aspirin group (*n* = 118)	*P* value
Gender (female/male)	81/71	62/56	1.00
Age (year)	60.17 ± 10.56	64.31 ± 7.96	*<0* *.* *001*
LBP history (month)	40.98 ± 62.51	48.55 ± 65.15	0.333
Alcohol consumption	62	52	0.677
Smoking	64	52	0.842
BMI (kg/m^2^)	25.97 ± 3.54	25.79 ± 3.4	0.669
VAS	5.56 ± 1.76	6.08 ± 1.72	*0*.*015*
CCI	1.94 ± 0.97	2.19 ± 1.07	*0*.*043*

LBP, low back pain; BMI, body mass index; VAS, visual analogue scale; CCI, Charlson Comorbidity Index; PSM, Propensity Score Matching.

Italics indicate statistically significant differences.

**Table 2:** Comparison of confounders after PSM.

	Control group (*n* = 73)	Aspirin group (*n* = 73)	*P* value
Gender (female/male)	37/36	32/41	0.606
Age (year)	62.57 ± 7.53	63.09 ± 7.48	0.690
LBP history (month)	45.15 ± 64.49	42.26 ± 50.10	0.771
Alcohol consumption	33	29	0.603
Smoking	34	30	0.604
BMI (kg/m^2^)	25.72 ± 3.27	25.70 ± 3.50	0.968
VAS	5.90 ± 1.64	5.85 ± 1.76	0.880
CCI	2.13 ± 0.99	2.13 ± 1.09	1.000

LBP, low back pain; BMI, body mass index; VAS, visual analogue scale; CCI, Charlson Comorbidity Index; PSM, Propensity Score Matching.

### MRI measurements

**Figure 7:**
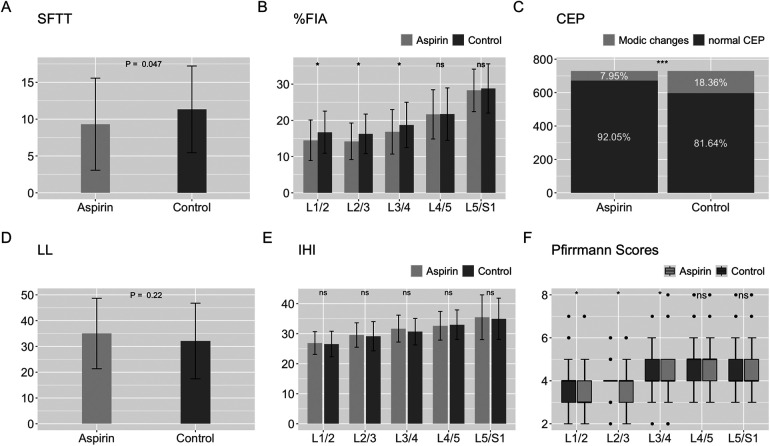
Statistical chart of SFTT, %FIA, CEP, LL, IHI, modified Pfirrmann grading scores. **(A)** Statistical chart of SFTT. **(B)** Statistical chart of %FIA. **(C)** Statistical chart of CEP. **(D)** Statistical chart of LL. **(E)** Statistical chart of IHI. **(F)** Statistical chart of modified Pfirrmann grading scores.

The mean SFTT(L1/2) in the aspirin group was 9.31 ± 6.25 mm, while it was 11.33 ± 5.89 mm in the control group (Table 3). For the paravertebral muscles %FIA from L1/2 to L5/S1, the aspirin group had averages of 14.49 ± 5.60, 14.22 ± 5.05, 16.63 ± 6.00, 21.65 ± 6.80, and 28.27 ± 5.86, respectively. In the control group, the corresponding %FIA values were 16.74 ± 5.81, 16.29 ± 5.45, 18.75 ± 6.24, 21.72 ± 7.23, and 28.80 ± 6.78 (Table 3). The incidence of CEP Modic changes was 7.95% in the aspirin group, compared to 18.36% in the control group (Table 4). When comparing the paravertebral muscles %FIA at the L4-S1 level between the aspirin and control groups, the differences were not statistically significant (*P* > 0.05). However, statistically significant differences were found between the aspirin and control groups in paravertebral muscles %FIA at the L1-L4 level, SFTT (L1/2), and the incidence of CEP Modic changes (*P* < 0.05) (Figures 7A–E).

**Table 3:** Comparison of paravertebral muscle %FIA and SFTT(L1/2).

	Aspirin group (*n* = 73)	Control group (*n* = 73)	*P* value
%FIA-L1/2	14.49 ± 5.60	16.74 ± 5.81	*0* *.* *019*
%FIA-L2/3	14.22 ± 5.05	16.29 ± 5.45	*0*.*019*
%FIA-L3/4	16.63 ± 6.00	18.75 ± 6.24	*0*.*038*
%FIA-L4/5	21.65 ± 6.80	21.72 ± 7.23	0.955
%FIA-L5/S1	28.27 ± 5.86	28.80 ± 6.78	0.615
SFTT (mm)	9.31 ± 6.25	11.33 ± 5.89	*0*.*047*

%FIA, fat infiltration area ratio; SFTT, subcutaneous fat tissue thickness.

Italics indicate statistically significant differences.

**Table 4:** Comparison of CEP pathological changes.

	Modic changes	Normal CEP	Total	*χ* ^2^	*P* value
Aspirin group (*n* = 73)	58 (7.95%)	672 (92.05%)	730 (100%)	34.64	*<0*.*01*
Control group (*n* = 73)	134 (18.36%)	596 (81.64%)	730 (100%)		

CEP, cartilage endplate.

Italics indicate statistically significant differences.

### Radiograph measurements

The mean LL in the aspirin group was 35.01 ± 13.66 degrees, while in the control group, it was 32.11 ± 14.66 degrees (Table 5). For the IHI from L1/2 to L5/S1, the aspirin group had averages of 26.60 ± 4.20, 28.68 ± 4.00, 30.38 ± 4.30, 33.06 ± 4.63, and 35.16 ± 6.05, while the control group had values of 25.95 ± 3.37, 28.54 ± 4.42, 30.96 ± 4.50, 31.91 ± 4.98, and 35.34 ± 7.83 (Table 5). Comparing the aspirin and control groups in terms of LL and IHI, the *p*-values were 0.22, 0.30, 0.84, 0.43, 0.15, and 0.76, respectively, indicating no statistically significant differences between the aspirin and control groups **(**Figures 7D,E).

**Table 5:** Comparison for LL and IHI.

	Control group (*n* = 73)	Aspirin group (*n* = 73)	*P* value
LL	35.01 ± 13.66	32.11 ± 14.66	0.2183
IHI-L1/2	26.60 ± 4.20	25.95 ± 3.37	0.3038
IHI-L2/3	28.68 ± 4.00	28.54 ± 4.42	0.8379
IHI-L3/4	30.38 ± 4.30	30.96 ± 4.50	0.4252
IHI-L4/5	33.06 ± 4.63	31.91 ± 4.98	0.1520
IHI-L5/S1	35.16 ± 6.05	35.34 ± 7.83	0.7601

LL, Lumbar lordosis; IHI, Intervertebral Height Index.

### Modified pfirrmann grading scores

From L1/2 to L5/S1, the median and IQR of the modified Pfirrmann grading scores in the aspirin group were as follows: 3 (IQR = 1.5), 4 (IQR = 1), 4 (IQR = 1.5), 5 (IQR = 1), and 4 (IQR = 1). The 95% confidence intervals (CI) for these scores were (3.05, 3.55), (3.53, 3.98), (3.79, 4.29), (4.42, 4.87), and (4.12, 4.62), respectively. In the control group, the median and IQR were 4 (IQR = 1), 4 (IQR = 0), 4 (IQR = 1), 5 (IQR = 1), and 4 (IQR = 1), with corresponding 95% CIs of (3.45, 4.00), (3.89, 4.25), (4.16, 4.55), (4.53, 5.09), and (4.17, 4.63) (Table 6). For L1/2, L2/3, and L3/4, the scores in the aspirin group were significantly lower than those in the control group, with *p*-values of 0.015, 0.024, and 0.013, respectively. However, for L4/5 and L5/S1, there were no statistically significant differences between the aspirin and control groups, with *p*-values of 0.514 and 0.607, respectively (Figure 7F).

**Table 6:** Comparison of modified Pfirrmann grading scores.

	Aspirin group (*n* = 73) vs. Control group (*n* = 73)				
	L1/2	L2/3	L3/4	L4/5	L5/S1
Grade 1	0/0	0/0	0/0	0/0	0/0
Grade 2	18/13	7/1	5/1	0/0	0/0
Grade 3	24/15	20/13	13/8	3/5	11/11
Grade 4	26/31	34/42	36/32	33/28	37/32
Grade 5	3/8	8/14	16/29	30/28	18/24
Grade 6	0/5	4/3	0/2	3/4	3/3
Grade 7	2/1	0/0	2/1	2/4	2/2
Grade 8	0/0	0/0	1/0	2/4	2/1
Median and IQR	3 (IQR = 1.5)	4 (IQR = 1)	4 (IQR = 1.5)	5 (IQR = 1)	4 (IQR = 1)
	4 (IQR = 1)	4 (IQR = 0)	4 (IQR = 1)	5 (IQR = 1)	4 (IQR = 1)
95% CI	(3.05, 3.55)	(3.53, 3.98)	(3.79, 4.29)	(4.42, 4.87)	(4.12, 4.62)
	(3.45, 4.00)	(3.89, 4.25)	(4.16, 4.55)	(4.53, 5.09)	(4.17, 4.63)
*Z* value	2.42	2.26	2.49	0.65	0.5
*P* value	*0.015*	*0.024*	*0.013*	0.514	0.607

IQR, interquartile range; CI, confidence interval.

Italics indicate statistically significant differences.

### Modified Pfirrmann grading scores for L1-4 vs. L4-S1

**Figure 8:**
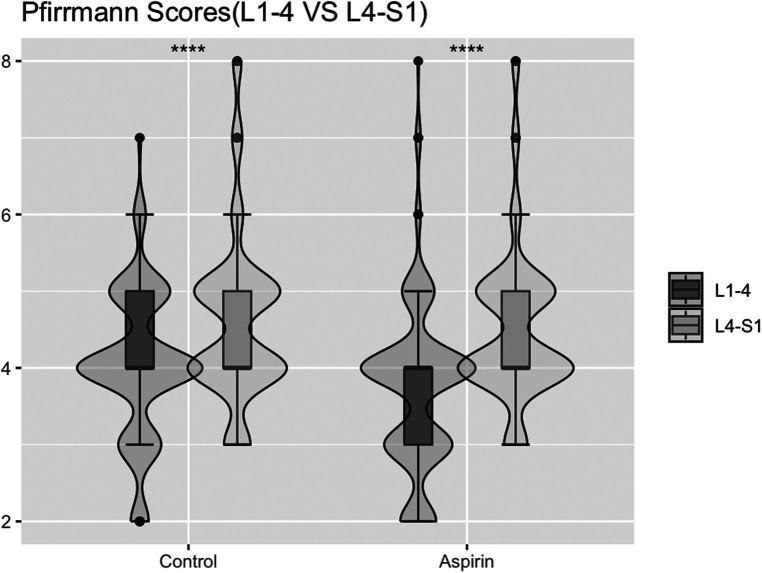
Statistical chart of comparison of upper lumbar (L1-4) vs. lower lumbar (L4-S1) by modified Pfirrmann grading scores.

In the control group, the median and IQR of the scores for the upper lumbar spine (L1-4) were 4 (IQR = 1), with a 95% CI of (3.92, 4.18), and for the lower lumbar spine (L4-S1), the median and IQR were also 4 (IQR = 1), with a 95% CI of (4.42, 4.78) (Table 7). In the aspirin group, the median and IQR for the upper lumbar spine (L1-4) were 4 (IQR = 1), with a 95% CI of (3.56, 3.84), similarly, for the lower lumbar spine (L4-S1), the median and IQR were 4 (IQR = 1), with a 95% CI of (4.34, 4.67) (Table 7). The scores for the upper lumbar spine were significantly lower than those for the lower lumbar spine (*P* < 0.05), indicating statistically significant differences (Figure 8).

**Table 7:** Comparison of modified Pfirrmann grading scores for L1-4 vs. L4-S1.

	Control group (*n* = 73)	Aspirin group (*n* = 73)
	L1-4 vs. L4-S1	L1-4 vs. L4-S1
Median and IQR	4 (IQR = 1)	4 (IQR = 1)
	4 (IQR = 1)	4 (IQR = 1)
95% CI	(3.92, 4.18)	(3.56, 3.84)
	(4.42, 4.78)	(4.34, 4.67)
*P* value	*<0.0001*	*<0.0001*

IQR, interquartile range; CI, confidence interval.

Italics indicate statistically significant differences.

## Discussion

This study aims to investigate the effect of aspirin on lumbar degeneration in patients with LBP and concurrent atherosclerosis using imaging-based assessments. To minimize the impact of confounding variables between the aspirin and control groups, we employed a multi-factor logistic regression model for PSM analysis, considering factors such as age, gender, smoking history, alcohol consumption, medical history, VAS scores, BMI, and CCI. After performing 1:1 matching of patients' data using PSM, we analyzed the differences between the aspirin and control groups regarding SFTT(L1/2), paravertebral muscles %FIA, LL, modified Pfirrmann grading scores, IHI, and the incidence of CEP Modic changes, to explore the effect of aspirin on lumbar degeneration. In this study, the aspirin group showed lower SFTT(L1/2) and a reduced incidence of CEP Modic changes, compared to the control group (both *P* < 0.05). Additionally, the %FIA and Pfirrmann scores were lower in the aspirin group, particularly in the upper lumbar spine (both *P* < 0.05). No significant differences were observed in LL and IHI between the aspirin and control groups.

West et al. found that SFTT(L1/2) was associated with individual obesity, which could predict LBP (17). Recent studies have indicated that SFTT(L1/2) correlates well with patients' general health status and can effectively predict both LBP and lumbar degeneration, demonstrating good sensitivity and specificity (18, 19). When the cutoff value for SFTT(L1/2) was set at 8.45 mm, the sensitivity and specificity were found to be 74% and 81%, respectively (19). The SFTT(L1/2) is positively correlated with the degree of degeneration. The thicker the fat layer, the higher the accuracy and specificity. For each 1 mm increase in SFTT(L1/2), the likelihood of experiencing LBP increases by 29% for women and 35% for men (19). Kanbayti et al. reported that females and younger individuals tend to have greater SFTT at the L1/2 level, which increases the risk of IVDD (15). This implies that increased subcutaneous fat in the back may contribute to the degeneration of lumbar discs. Poot et al. demonstrated that the average lumbar SFTT is associated with the overall occurrence of disc herniation, noting that more severe herniations correspond to higher SFTT values, thus indicating that SFTT is a reliable predictor of disc degeneration (20). Our study subjects were patients with symptomatic LBP. The SFTT(L1/2) of the aspirin group was 9.31 ± 6.25 mm, and the SFTT(L1/2) of the control group was 11.33 ± 5.89 mm. Both groups were higher than 8.45 mm. The SFTT(L1/2) of the aspirin group was significantly lower than that of the control group, the differences were statistically significant (*P* = 0.047), indicating that the degree of lumbar degeneration in the aspirin group was milder than that of the control group.

The paravertebral muscles play an important role in maintaining the structural stability and function of the lumbar spine (21). There is a reciprocal relationship between lumbar degeneration and paravertebral muscle degeneration. Degeneration of the paravertebral muscles can lead to lumbar instability, which may cause or worsen lumbar spine degeneration, while lumbar degeneration can further exacerbate paravertebral muscle degeneration (22). Khattab et al. observed that fat content increases in degenerative muscle tissue, and the area of fat infiltration in the paravertebral muscles, as quantified by lumbar cross-sectional MRI, is often used to evaluate paravertebral muscle mass (23). The %FIA is the most commonly employed method (24). The paravertebral muscles %FIA is related to lumbar degeneration, and the more severe the lumbar degeneration, the larger the paravertebral muscles %FIA (25). Huang et al. reported a correlation between the paravertebral muscles' %FIA and the severity of disc degeneration at each intervertebral space (26). Shen et al. found that fat infiltration in the paravertebral muscles was linked to multi-level lumbar disc degeneration, identifying it as an independent risk factor for multi-level degeneration in patients with lumbar disc herniation (22). Chen et al. noted a weak positive correlation between paravertebral muscles %FIA and lumbar facet joint degeneration, while a strong correlation was found between the degree of fat infiltration in patients with chronic LBP and IVDD (27). In our study, the %FIA in the L1-4 intervertebral disc segment was significantly lower in the aspirin group compared to the control group (*P* < 0.05), suggesting that the paravertebral muscle degeneration in the L1-4 segment of the aspirin group was less severe than that in the control group. Although there were no significant differences in the %FIA for the corresponding segment of the L4-S1 intervertebral disc between the aspirin and control groups, the mean ± standard deviation for both L4-5 and L5-S1 in the aspirin group was lower than in the control group. The lack of statistically significant differences may be attributed to the small sample sizes in both groups and a larger sample size might reveal more significant statistical differences. Huang et al. also found that paravertebral muscles %FIA was influenced by gender and age, with females exhibiting higher levels than males, and %FIA increasing with age (26). However, our study controlled for age and gender differences between the aspirin and control groups through PSM. Greg et al. discovered that TNF expression was higher in the paravertebral muscles of patients with high-fat infiltration through genetic testing, suggesting that the increased %FIA in paravertebral muscles may be linked to inflammatory disorders in degenerative spinal muscles (28). A review indicated that heightened inflammation is likely a significant factor in promoting fat infiltration in skeletal muscle and that NSAIDs can help reduce %FIA in muscles (29). This suggests that the anti-inflammatory effect of aspirin may contribute to the observed differences in %FIA between the aspirin and control groups, although this remains indirect evidence.

A study indicated that more severe IVDD is associated with a smaller LL (12). Chun et al. conducted a review showing that patients with LBP have a smaller LL compared to healthy individuals (30). Another prospective study revealed a link between the occurrence of LBP and reduced LL (31). As individuals age, lumbar degeneration typically worsens, which corresponds with a decrease in LL (32). In our findings, the LL measured 35.01 ± 13.66 degrees in the aspirin group and 32.11 ± 14.66 degrees in the control group. The mean LL in the aspirin group was higher than in the control group, these differences were not statistically significant (*P* = 0.2183), which may be due to the small sample size.

The Modified Pfirrmann grading system is a simple, practical, and reproducible method and the most commonly used method to assess the grade of disc degeneration (14). Our study found that the modified Pfirrmann grading scores for the L1/2, L2/3, and L3/4 intervertebral discs in the aspirin group were lower than those in the control group, with *p*-values below 0.05. This indicates that the aspirin group had milder disc degeneration compared to the control group. There were no significant differences in the modified Pfirrmann grading scores for the L4/5 and L5/S1 intervertebral discs, as *p*-values for these comparisons exceeded 0.05, suggesting that the grade of disc degeneration in these segments was similar between the aspirin and control groups. When defining L1-4 as the upper lumbar spine and L4-S1 as the lower lumbar spine (33), we observed that the modified Pfirrmann grading scores for the upper lumbar spine were lower than those for the lower lumbar spine in both groups. This finding suggests that the upper lumbar spine exhibits significantly less degeneration compared to the lower lumbar spine in aspirin and control groups. Several factors contribute to degeneration in the lower lumbar spine (L4-S1), such as the greater shear forces experienced by the L4-S1 intervertebral disc, leading to more severe degeneration that may necessitate surgical intervention instead of conservative treatment. From a biomechanics perspective, the L4-S1 intervertebral disc experiences the highest pressure and greatest movement. Additionally, the posterior longitudinal ligament behind these two segments is relatively narrow, about half the width of the ligaments in the discs above. As a result, herniation of the L4-S1 disc tends to be more severe and progresses more rapidly. We hypothesize that the protective effect of aspirin on the lower lumbar spine was inadequate to counteract the influence of these other factors promoting disc degeneration, resulting in continued degeneration in the L4-S1 segment for both groups and masking the protective effect of aspirin.

The IHI is an indicator used to evaluate the loss of intervertebral disc height (34). In assessing lumbar degeneration, it serves as a supplementary measure to the modified Pfirrmann grading system (35). We found that the IHIs for L1-S1 in the aspirin group were 26.60 ± 4.20, 28.68 ± 4.00, 30.38 ± 4.30, 33.06 ± 4.63, and 35.16 ± 6.05, while those in the control group were 25.95 ± 3.37, 28.54 ± 4.42, 30.96 ± 4.50, 31.91 ± 4.98, and 35.34 ± 7.83. The *p*-values for these comparisons were all greater than 0.05, indicating that the differences were not statistically significant. However, the values for the aspirin group were generally higher than those for the control group, suggesting that aspirin had a certain degree of protective effect against IHI. According to our statistical chart (Figure 7F), over 75% of the modified Pfirrmann grading scores for the intervertebral discs in both groups were lower than grade 5, with grade 5 indicating no significant reduction in intervertebral disc height. This consistency suggests that the degree of intervertebral disc height degeneration was similar between the aspirin and control groups, with the evaluations of IHI and Pfirrmann grades aligning.

Vertebral endplate osteochondritis, or Modic changes, refers to aseptic inflammation of the CEP, resembling the subchondral osteosclerosis seen in osteoarthritis. Modic changes are more likely to occur in the lumbar spine and are considered one of the causes of LBP in patients (25). The function of the CEP is to withstand pressure, protect the vertebral body, and control the nutrient penetration of the intervertebral disc. CEP injury causes disc degeneration, also a predisposing factor for CEP Modic changes (36, 37). Karchevsky et al. found that Modic changes were more prevalent in the L4/5 and L5/S1 segments, with patients suffering from LBP showing Modic changes seven times more frequently than asymptomatic individuals (43% vs. 6%) (38). Modic changes are also seen as indicative of lumbar degeneration. Rodrigues et al. established that Modic changes correlate with the grades of the modified Pfirrmann grading system, with more severe disc degeneration closely related to both the modified Pfirrmann grading system and Modic classification (39). Mok et al. noted a connection between Modic changes and the presence and severity of disc degeneration and LBP (40). Risk factors for chondral endplate inflammation include disc degeneration, age, obesity, and others (40, 41). Gualdi et al. found that Modic changes were linked to obesity and the %FIA in the paravertebral muscles (41). In our study, the incidence of CEP Modic changes was 7.95% in the aspirin group and 18.36% in the control group (*P* < 0.01), revealing a statistically significant difference. This suggests that the aspirin group had a significantly lower incidence of CEP Modic changes compared to the control group. The results for the modified Pfirrmann grading system scores, %FIA, SFTT(L1/2), and Modic changes were consistent between the aspirin and control groups, indicating that aspirin serves as a protective factor against lumbar degeneration.

However, our study has limitations. First, being a retrospective imaging-based case-control study, it does not provide the same level of evidence as a prospective cohort study. Second, as a single-center study with a small sample size, we aim to further explore the effects of aspirin on lumbar degeneration through a multicenter, large-sample prospective study. Lastly, since our results are based on patients with carotid plaques or atherosclerotic cardiovascular or cerebrovascular diseases, further research is needed to determine whether these findings apply to broader populations.

## Conclusions

Conservative treatment with aspirin exerts a protective effect against degeneration in the upper lumbar spine, while its protective effect on the lower lumbar spine is less pronounced.

## Data Availability

The data are not publicly available due to their containing information that could compromise the privacy of research participants. Requests to access the datasets should be directed to Haiyun Niu, niuhaiyun@hebmu.edu.cn.
